# Fossil evidence for a pharyngeal origin of the vertebrate pectoral girdle

**DOI:** 10.1038/s41586-023-06702-4

**Published:** 2023-11-01

**Authors:** Martin D. Brazeau, Marco Castiello, Amin El Fassi El Fehri, Louis Hamilton, Alexander O. Ivanov, Zerina Johanson, Matt Friedman

**Affiliations:** 1 Department of Life Sciences, Imperial College London, Ascot, UK; 2 The Natural History Museum, London, UK; 3 Department of Sedimentary Geology, Institute of Earth Sciences, St Petersburg State University, St Petersburg, Russia; 4 Institute of Geology and Petroleum Technologies, Kazan Federal University, Kazan, Russia; 5 Museum of Paleontology, University of Michigan, Ann Arbor, MI USA; 6 Department of Earth and Environmental Sciences, University of Michigan, Ann Arbor, MI USA; 7 Present Address: London Academy of Excellence, London, United Kingdom; 8 Present Address: Paläontologisches Institut und Museum, Universität Zürich, Zurich, Switzerland

**Keywords:** Palaeontology, Phylogenetics

## Abstract

The origin of vertebrate paired appendages is one of the most investigated and debated examples of evolutionary novelty^1–7^. Paired appendages are widely considered as key innovations that enabled new opportunities for controlled swimming and gill ventilation and were prerequisites for the eventual transition from water to land. The past 150 years of debate^8–10^ has been shaped by two contentious theories^4,5^: the ventrolateral fin-fold hypothesis^9,10^ and the archipterygium hypothesis^8^. The latter proposes that fins and girdles evolved from an ancestral gill arch. Although studies in animal development have revived interest in this idea^11–13^, it is apparently unsupported by fossil evidence. Here we present palaeontological support for a pharyngeal basis for the vertebrate shoulder girdle. We use computed tomography scanning to reveal details of the braincase of *Kolymaspis sibirica*^14^, an Early Devonian placoderm fish from Siberia, that suggests a pharyngeal component of the shoulder. We combine these findings with refreshed comparative anatomy of placoderms and jawless outgroups to place the origin of the shoulder girdle on the sixth branchial arch. These findings provide a novel framework for understanding the origin of the pectoral girdle. Our evidence clarifies the location of the presumptive head–trunk interface in jawless fishes and explains the constraint on branchial arch number in gnathostomes^15^. The results revive a key aspect of the archipterygium hypothesis and help reconcile it with the ventrolateral fin-fold model.

## Abstract (web summary)

Computed tomography analysis of the braincase of the Early Devonian placoderm fish *Kolymaspis sibirica* suggests a skeletal gill support was involved in the origin of the shoulder girdle and provides new evidence reconciling historic theories about the evolution of paired fins.

## Main

The two major theories of the origin of vertebrate appendages differ in their ability to explain evolutionary patterns. The ventrolateral fin-fold hypothesis proposes that paired fins arose from ventrolateral keels extending the length of the trunk, which became subdivided into pectoral and pelvic fins. The archipterygium hypothesis argues that the girdles derived from an ancestral skeletal gill arch and that the fin endoskeleton formed from gill rays. The fin-fold hypothesis is seen as the more ‘successful’ of the two theories^4,16^, with support from developmental genetics^17^ and widespread evidence of stem-group gnathostomes possessing ventrolateral fin folds in some form^5,7^. However, the fin-fold hypothesis does not explain the origin of the pectoral girdle, which resulted in the subdivision of the head into a separate skull and shoulder. Furthermore, it predicts the simultaneous origin of pectoral and pelvic fins, which is currently contradicted by fossil data^5,7^. The archipterygium hypothesis explains the pectoral girdle and separate origins of pectoral and pelvic fins by basing their origins on pre-existing structures. Clues from developmental genetics^11–13^ have renewed interest in the archipterygium hypothesis as a viable theory.

A key challenge to testing the archipterygium hypothesis with evidence from the fossil record is the rarity of fossilized gill arches. Gill arches are cartilage-derived endoskeletal structures that were either unossified or weakly ossified and are therefore not preserved in the earliest fossil taxa. The closest fossil sister group of jawed vertebrates is the Osteostraci, which ranges from the Wenlock epoch of the Silurian period to the Late Devonian period (approximately 432 to 378 million years ago (Ma)). Osteostracans possessed distinct pectoral fins, but these were attached to a unified craniothoracic block of cartilage that was surmounted by tessellated dermal bone. Fossilized pharyngeal arches are completely unknown in osteostracans, obscuring reconstructions of pharyngeal conditions^18^ preceding the origins of jaws and a pectoral girdle. The phylogenetically earliest jawed vertebrates are the placoderms, heavily armoured predatory fishes and contemporaries of osteostracans. Placoderm gill arches are rarely preserved and incompletely understood^19,20^. However, a crucial piece of evidence has long been overlooked. Despite the rarity of the arches themselves, their attachments are well-preserved as discrete facets near the perimeter of well-ossified braincases of both osteostracans and placoderms^18^. In osteostracans, however, the articulation facets are quite remote from the core of the braincase, situated near the perimeter of a broad cephalic shield that defines an enlarged oralobranchial chamber (see below). Alongside these are well-established anatomical landmarks in the form of cranial innervation and blood supply patterns, recorded as grooves or ossified canals within these braincases and consistent across vertebrates. This record of both hard and inferred soft-tissue anatomy provides a framework for investigating the role of the pharynx in the skeletal and bodyplan transformations leading to the origin of paired pectoral fins and a distinct pectoral girdle.

**Fig. 1:**
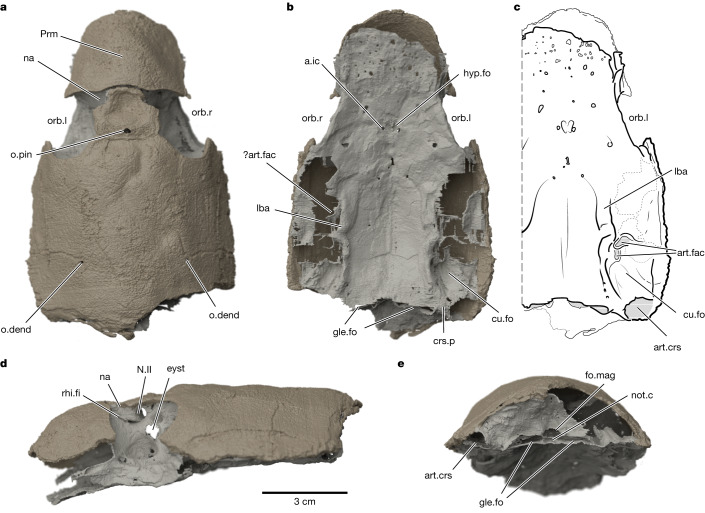
The braincase and skull roof of *K. sibirica* Bystrow 1956 specimen TsNIGR 7656 as a virtual three-dimensional rendering. **a**, Dorsal view. **b**, Ventral view. **c**, Interpretive illustration of ventral view. **d**, Left lateral view. **e**, Posterior view. a.ic, foramen for internal carotid artery; art.crs, articular facet on end of craniospinal process; art.fac, articular facets for branchial arches; crs.p, craniospinal process; cu.fo, cucullaris muscle fossa; eyst, eystalk attachment; fo.mag, foramen magnum; gle.fo, fossa for occipital glenoid facets; hyp.fo, hypophyseal fossa; lba, laterobasal angle; N.II, optic tract canal; na, naris; not.c, notochordal canal; o.dend, endolymphatic duct opening; o.pin, pineal opening; orb.l, left orbit; orb.r, right orbit; Prm, premedian plate; rhi.fi, rhinocapsular fissure. Dark beige material is dermal (exoskeletal) bone and light beige material is perichondral (endoskeletal) bone.

**Fig. 2:**
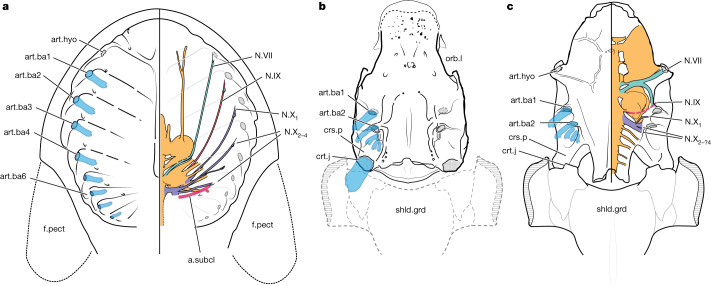
Comparative anatomy of cranial processes and branchial arch attachments in stem gnathostomes. **a**, Osteostracan *Nectaspis* (composite based on ref. ^47^). **b**, Acanthothoracid placoderm *Kolymaspis*. **c**, Acanthothoracid placoderm *Romundina* (original based on data from ref. ^48^ and new data). Transparent blue structures represent reconstructed branchial arches. art.ba1–6, serially numbered branchial arch attachments (corresponds to art.fac in Fig. 1); art.hyo, hyoid arch articulation; a.subcl, canal for subclavian artery; crt.j, craniothoracic joint; f.pect, pectoral fin; N.VII, facial nerve canal; N.IX, glossopharyngeal canal; N.X_1–4_, vagus nerve canal branches (numbered 1–4); shld.grd, shoulder girdle. Not to scale.

**Extended Data Fig. 1:**
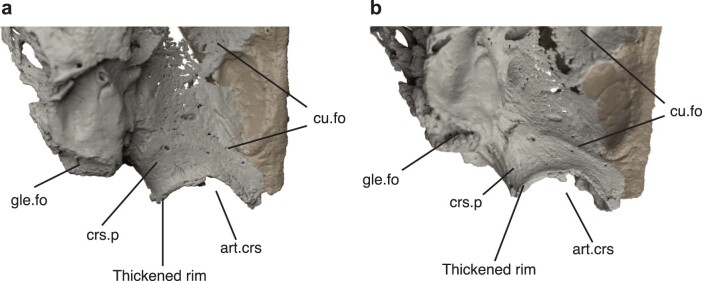
Craniospinal process area of *Brindabellaspis* (Australian National University specimen 49493) showing the structure of the articular facet. **a**, Ventral view. **b**, Oblique posteroventral view. Abbreviations as in Fig. 1. Original segmentation of data from (ref. ^26^). Colour convention as in Fig. 1 (Main Text).

The type and only specimen of *K. sibirica* (FN Chernyshev Central Research Geological Museum in St Petersburg, Russia, TsNIGR 7656) is a three-dimensionally preserved skull roof and braincase (Fig. 1). The skull of *Kolymaspis* is anteroposteriorly elongate, with a pronounced premedian ‘snout’ (upper lip, sensu ref. ^21^) and large, dorsolaterally directed orbits. The dermal skull roof is nearly complete, with the separately ossified rostropineal plate and rhinocapsular ossification in articulation (Fig. 1). The dermal skull roof is coated in stellate tubercles, consistent with ‘acanthothoracid’ placoderms^22–24^. In ventral view, the braincase is broad and deeply concave; the parachordal region is laterally demarcated by raised longitudinal crests (laterobasal angles; Fig. 1). The parachordal plates here terminate posteriorly without forming a marked occipital process; the occipital glenoid facets (attachments to the spinal column) were flush with the posterior margin of the braincase, in a condition similar to *Brindabellaspis*^25,26^. They are wide, dorsoventrally flat, openings flanking the notochordal canal. Posteriorly, the braincase flares laterally into stout craniospinal processes (Fig. 1). This process is most complete on the left (observer right) side. The distal part of the craniospinal process is an open, rimmed facet (Fig. 1), indicating it was the articulation point for a second cartilage. This corresponds with *Brindabellaspis*, which also has a terminal facet on the craniospinal process^25^ (Extended Data Fig. 1). However, this feature is unknown in any other placoderms, in which the facet is either absent and the craniospinal process is wholly covered in perichondral bone (as in *Romundina*; Fig. 2c), or capped in dermal bone as in arthrodires. In posterior view, the occipital surface also resembles *Brindabellaspis* in being broad, centrally concave and lacking identifiable cavities for paired epaxial musculature (muscles raising the skull). The foramen magnum is nearly twice the diameter of the notochordal canal, consistent with stem-group gnathostome conditions, and the two are contiguous openings positioned near the ventral margin of the braincase (Fig. 1).

These observations of the *Kolymaspis* braincase and comparisons to other taxa enable us to identify the ancestral position of head–shoulder separation in jawless fishes and propose specific musculoskeletal transformations in the origin of the gnathostome pectoral girdle. The placoderm craniospinal processes articulate with the pectoral girdle (shld.grd; Fig. 2). The open articular facet on the craniospinal process of *Kolymaspis* and *Brindabellaspis* (hereafter referred to collectively as brindabellaspidids^25^) points to an endoskeletal element here, forming a junction with the pectoral girdle. This is notable because this endoskeletal element would lie in series with the pharyngeal arches (Fig. 2) and a key anatomical landmark of the head–shoulder boundary in gnathostomes: the cucullaris muscle, responsible for depressing the skull towards the shoulder girdle^27–29^. There is a wealth of anatomical and developmental evidence that the cucullaris muscle is of branchial origin^16,30–32^. This gives rise to the prediction that it may have ancestrally joined a branchial arch. We propose that this endoskeletal element is a serial homologue of an upper branchial element (epibranchial or pharyngobranchial, given its topological position) and therefore that the shoulder girdle of these taxa incorporated the dorsal element of a gill arch. Although placoderm braincases possess only two clear articular facets for branchial arches, rare skeletal material shows that they possess at least five skeletal arches (the posteriormost arch may be specialized, as in some chondrichthyans)^20^. No placoderms are known to possess more than this number of arches. This anatomical interpretation implies that the sixth branchial arch would most probably have been the one incorporated into the pectoral girdle, if our interpretation is correct.

**Extended Data Fig. 2:**
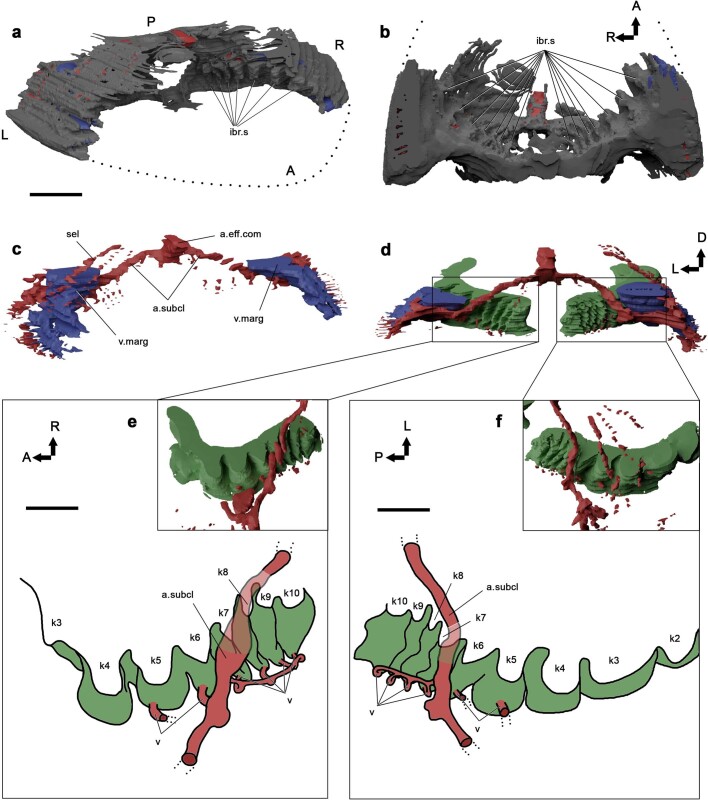
Posterior section of the skull and endocast of the osteostracan *Mimetaspis hoeli* showing the position of the subclavian artery relative to the gill compartments. **a**, Skull. **b**, Ventral view of the skull. **c**, Endocast of blood vessels and ‘sel’ canals. **d**, Posterior view of the endocast and branchial space. **e**, Dorsal view of the left side arteries and branchial space. **f**, Dorsal view of the right side arteries and branchial space. **a** and **c** are from the same view. Additional abbreviations: a.eff.com, common efferent artery (dorsal aorta); ibr.s, interbranchial septa; k2–k10, pharyngeal cavities; sel, sensory field canals; v, canals for vessels; v.marg, marginal vein cavities. Colour scheme: dark grey, bone; red, small canals; blue, large canals and cavities; green, branchial space. Dotted lines indicate where the continuation of the specimen would be if it was complete. The arrows indicate the orientation of the specimen: A, anterior; D, dorsal; L, left; P, posterior; R, right. Top left scale bar is for **a**, **b** and **c** and equals 6 mm. Bottom left scale bar is for **e** and equals 3 mm. Bottom right scale bar is for **f** and equals approximately 4 mm.

The sixth branchial arch is key in comparisons with jawless outgroups and enables independent support of our topological observations. Osteostracans differ from all known jawed vertebrates in the absence of a distinct head–shoulder separation, which is generally regarded as the ancestral gnathostome condition^27,28^. There are also no obvious points of homology that mark this separation in osteostracans. However, our hypothesis locates this presumptive division at the level of the sixth branchial arch. Notably, osteostracans frequently preserve a canal for the subclavian artery, the main arterial branch that supplies the pectoral fins. This artery stems from a cluster of arteries supplying the most posterior efferent branchial arteries serving the posterior pharyngeal arches^18,33,34^. The main trunk of the subclavian artery is seen in several specimens, showing that it extends along the interbranchial ridge of the sixth and seventh branchial arches^33,34^ (Fig. 2a and Extended Data Fig. 2). Locating the shoulder on the sixth branchial arch also provides a precise explanation for a puzzling phenomenon in which most gnathostomes appear to be constrained to no more than five gill arches (hexanchiform sharks notwithstanding—these appear to involve duplication of an intermediate arch^35^), whereas jawless fishes range from five to several dozen separate gill compartments^15^. If the ancestral pectoral girdle incorporated the sixth branchial arch, this would strongly bias the standard complement of jawed vertebrate arches to no more than five.

**Fig. 3:**
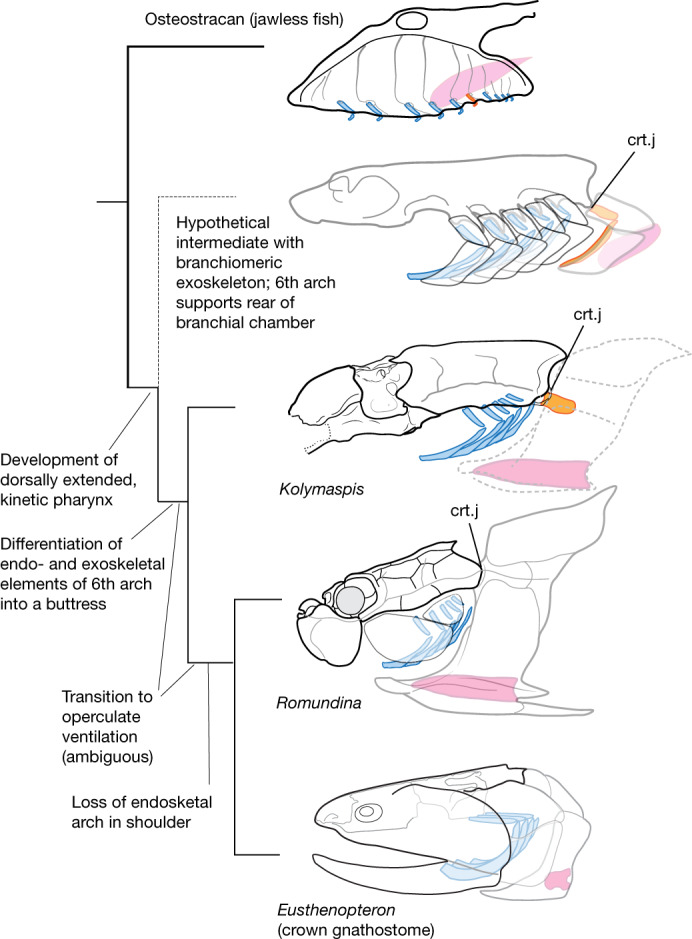
Summary phylogeny of early gnathostomes with reconstructions to show comparative anatomy of pharyngeal arches and shoulder linkages. Hypothetical intermediate is shown, for clarity of comparative anatomy; specific geometries may have varied substantially. Gill arch morphologies in osteostracan and placoderms are hypothetical and are shown to indicate location of articulations and constraints on overall pharynx architecture. Blue, branchial arches; orange, sixth branchial or thoracic arch; pink, pectoral fin attachment or scapulocoracoid. See Supplementary Information for complete phylogeny. Dashed lines indicate inferred pectoral girdle. Osteostracan is a composite based on ref. ^47^; *Romundina* is based on ref. ^49^ and new data; *Eusthenopteron* is a composite based on ref. ^50^.

**Extended Data Fig. 3:**
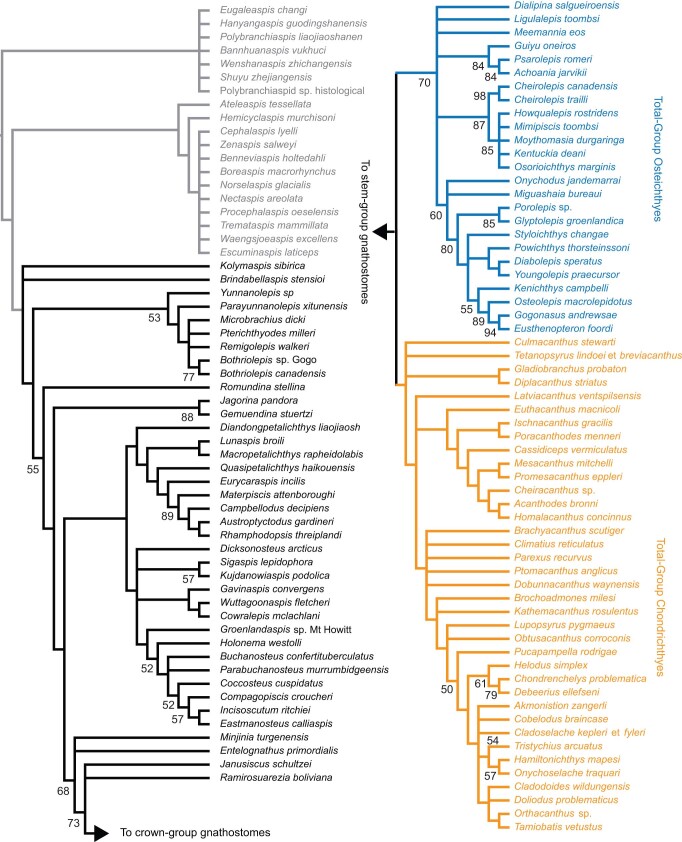
Strict consensus tree from equal weights parsimony analysis. Outgroup (jawless vertebrates) shown in light grey. See supplementary information for details of tree number and tree score. Numbers below nodes show bootstrap values 50% or above.

**Extended Data Fig. 4:**
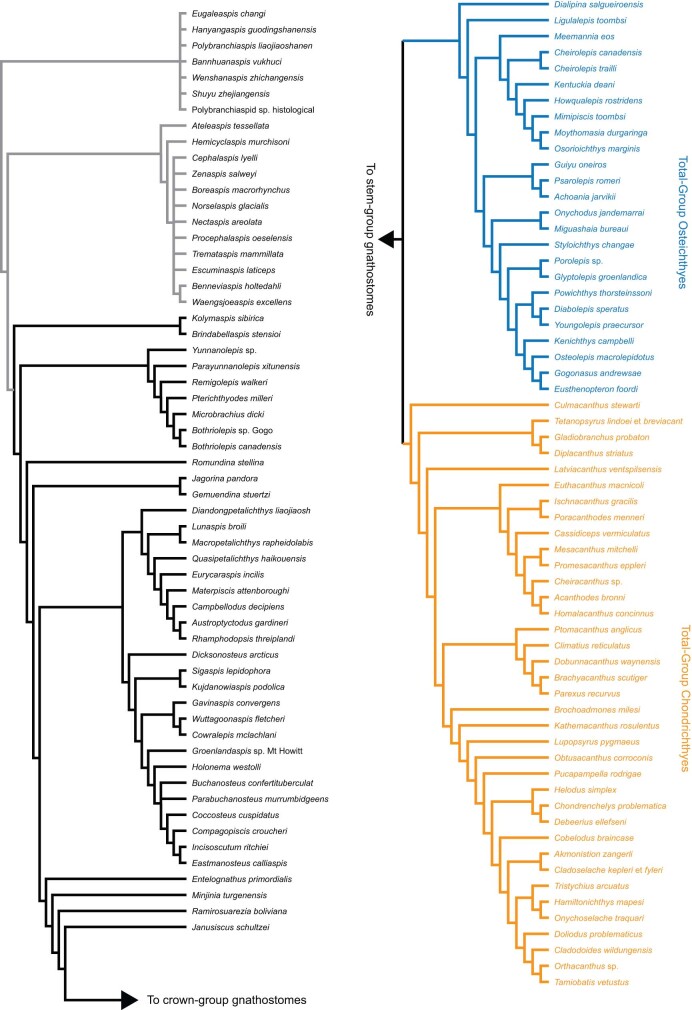
Strict consensus tree from implied weights (*k* = 12) parsimony analysis. Outgroup (jawless vertebrates) shown in light grey. See supplementary information for details of tree number and tree score.

**Extended Data Fig. 5:**
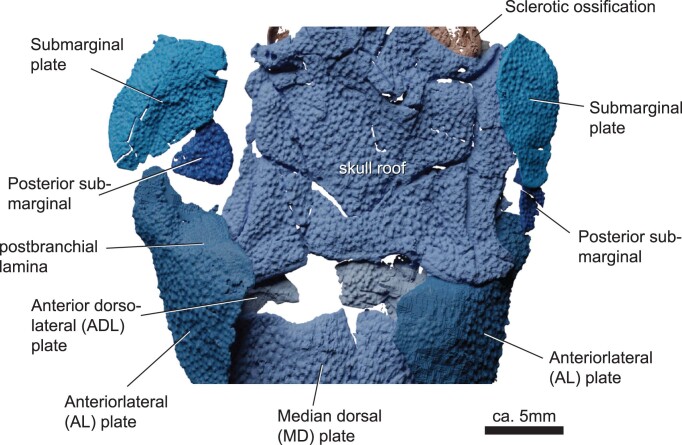
*Romundina* sp. specimen GZG 100–488A showing multiple bony elements in opercular series .

These observations in a phylogenetic context (Fig. 3 and Extended Data Figs. 3 and 4) enable us to propose a new hypothesis for the origin of the pectoral girdle. We propose that the pectoral girdle is established on the position of the sixth branchial arch in the jawless ancestor of jawed vertebrates, and that this structure formed the primary basis of a separate head and shoulder. The initial incorporation of the gill arch provided support for the rear wall of the pharynx, joined to the skull by a kinetic, moveable linkage (Fig. 3). This link persisted in placoderms as a craniothoracic joint, and in some taxa (such as the brindabellaspidids) a vestige of the endoskeletal component remained (Fig. 3). In modern gnathostomes, the endoskeletal elements of this sixth branchial arch are completely lost (see next paragraph on origin of scapulocoracoid). However, exoskeletal (dermal) components of the pectoral girdle (for example, cleithrum and clavicle) may have their origins from branchiomeric dermal plates covering this arch (that is, from a branchial operculum). Evidence from *Romundina* indicates that some placoderms possessed dermal branchial coverings posterior to the submarginal plate, which is the main opercular bone in placoderms (Extended Data Fig. 5). A similar condition is possible in the enigmatic new taxon *Xiushanosteus* from the early Silurian of China^36^. If one reinterprets the larger, more posterior post-suborbital plate in *Xiushanosteus *as a submarginal, then the smaller plate originally identified as a submarginal becomes a posterior submarginal similar to *Romundina*.

We can tentatively suggest new points of homology between the heads of osteostracans and jawed vertebrates and suggest specific skeletal transformations that occurred during the origin of the pectoral girdle. First, the postbranchial lamina (rear wall of the gill chamber) is a putative homologue of a plate of branchial association (Fig. 3); this is consistent with its position and demonstrated ability to support development of tooth-like denticles^37^, suggesting that it at least partly derives from cranial neural crest^28^. The formation of a postbranchial lamina occurred as the gill openings changed from pore-like openings of jawless fishes into deep-sided clefts of jawed taxa. This was concomitant with changes to the structure of the braincase, in which the broad lateral brim was withdrawn medially, exposing the expanded clefts laterally. The sixth arch lost respiratory tissue (gills) and became the basis of a craniothoracic joint supporting feeding or buccal pumping. We do not necessarily invoke the gill arches as the anatomical precursor of the pectoral fin skeleton or the scapulocoracoid, as predicted by the archipterygium hypothesis. Recent fate-mapping studies in skate (Chondrichthyes) show that the scapulocoracoid is composed of trunk mesoderm^13^ (compared to a zone of mixed cranial neural crest and trunk mesoderm in the gill arches). Additionally, a pectoral fin and proximal attachment was already present and anatomically separate from the gill arches in the osteostracans. Gill arches and pectoral elements thus fail the conjunction test of homology. Nevertheless, the close anatomical proximity of these structures would have allowed them to join a common dermal support (Fig. 3).

Our hypothesis partly revives the archipterygium hypothesis, but not as originally envisioned by Gegenbaur^8^. There is no known fossil evidence of a direct skeletal remnant of the ancestral gill arch in crown-group gnathostomes, only traces in the form of patterns of vascularization and musculature inherited from jawless ancestors^28^. Notably, the last direct vestiges of a pharyngeal arch in the shoulder girdle would have been lost in placoderms (Fig. 3), with evidence seen only in the enigmatic brindabellaspidids as described above. There is no requirement in our hypothesis, however, for either the scapulocoracoid or the pectoral fin endoskeleton to be of pharyngeal origin, as in Gegenbaur’s archipterygium. A separate head–girdle instead evolved as part of changes in the architecture of the pharynx, rather than primarily to support fins. Our work arrives independently at a previous suggestion that the pectoral girdle and fin are a morphological amalgam of cranial and thoracic regions of the body^16^. Fossil evidence for this has previously been suggested by Zangerl^38^. However, as with Gegenbaur’s original theory, Zangerl’s idea relied heavily on chondrichthyan anatomy^38^, referencing symmoriids and iniopterygians. These taxa are increasingly demonstrated as highly nested within chondrichthyans and well removed from the origin of gnathostomes^39,40^, casting doubt on their value as models for ancestral jawed vertebrates. Thus, elements of both the archipterygium and the fin-fold hypotheses are combined to explain the origin of pectoral appendages, the shoulder and a distinct gnathostome head as a total system. All these conclusions could potentially be tested by fate-mapping studies in modern osteichthyans (as these taxa retain the dermal pectoral girdle) as well as through new fossil finds of early gnathostomes.

This interpretation adds important functional details to the tight phylogenetic connection between the origin of a pectoral girdle and the origin of jaws. The craniospinal process is one of the pivot points in the four-bar linkage that makes up the placoderm jaw-closing apparatus^41^. This suggests that as a sixth branchial arch became established as the rearmost support and a kinetic joint, tying the origin of the pectoral girdle to a suite of changes to the pharynx involved in opening and closing the mouth and throat. Recent evidence suggests a compact, operculate pharynx as the ancestral condition for gnathostomes^42^, rather than the historically accepted shark-like septate model. Thus, it is reasonable to conclude that the origin of the pectoral girdle is integrated with the evolution of a compact bucco-pharyngeal apparatus for efficient gill ventilation or feeding.

**Extended Data Fig. 6:**
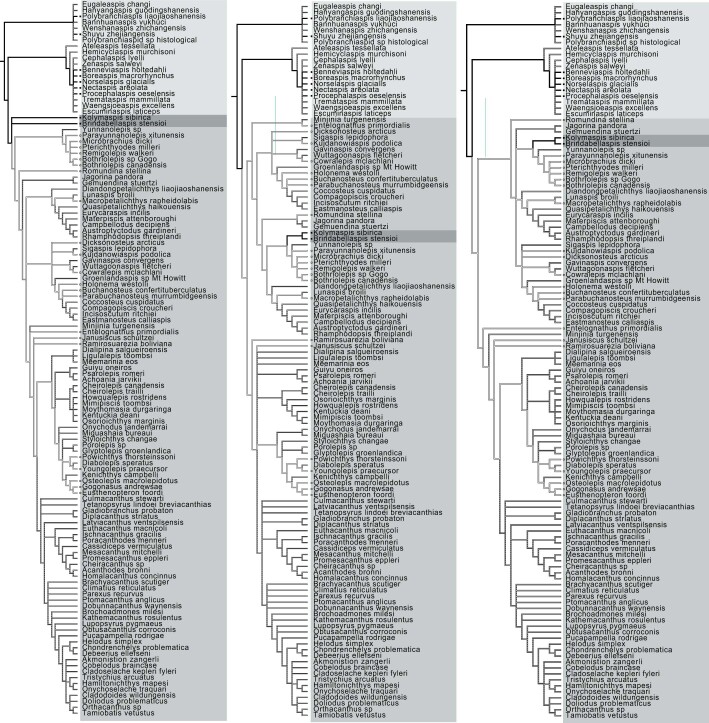
Gnathostome phylogeny under two constraints on placoderm monophyly. Left, strict consensus tree of equally weighted parsimony analysis.Centre, all placoderms inclusive of *Entelognathus* and *Minjinia* constrained as a clade; Right, “core placoderms” (excluding *Entelognathus* and *Minjinia*) constrained as a clade. Colour coding shows parsimony ancestral states mapping for character 180 (Endoskeletal craniothoracic [sixth branchial] facet). Black: present; white: absent; gray: ambiguous.

Our hypothesis is testable on several lines of evidence that could eventually overturn it. We discuss these along with existing points of weakness. First, it depends on the resolution of either *Kolymaspis* or *Brindabellaspis* as the sister group taxa of all other jawed vertebrates, and thus rests on the hypothesis of placoderm paraphyly. This is currently the case in our phylogeny (Extended Data Fig. 6). However, statistical support for placoderm paraphyly is weak (see bootstrap values in Extended Data Fig. 3) and highly debated^43,44^. Under placoderm monophyly, our hypothesis depends at least on the phylogenetic mapping of the craniothoracic facet to the base of all jawed vertebrates. We conducted additional analyses with constraints on placoderm monophyly leading to equivocal support for our new hypothesis (Extended Data Fig. 6 and Supplementary Information). Thus, new phylogenetic tests could reveal that the condition in the brindabellaspidids is uniquely derived (that is, neomorphic). The discovery of new fossils with both supernumerary branchial arches and a discrete pectoral girdle would also challenge our hypothesis. Furthermore, an alternative interpretation of the articular facet in brindabellaspidids is that it represents a connection to a shoulder cartilage not of pharyngeal origin. In our view, these explanations are less parsimonious and do not help account for the branchiomeric derivation of the cucullaris muscle, but they could be supported by future fossil discoveries or phylogenetic analyses. Even if those specifics are rejected, our hypothesis adds important new comparative anatomical perspectives that better reconcile the disparate anatomies of osteostracans and placoderms.

Our proposal synthesizes findings from the past two decades of research into the origin of the pectoral girdle. Furthermore, it clarifies key questions of comparative anatomy that have impeded studies on the origin of the vertebrate neck and shoulders. Key among these is resolution of the identity and location of the cucullaris muscle in osteostracans, a crucial anatomical landmark in establishing the head–shoulder interface^27–29,45^. Previous studies have struggled to identify the location of the cucullaris in osteostracans, concluding that it was absent^27^ or placing it in an epaxial location^46^. We argue that it was an undifferentiated branchial levator or protractor muscle and would have been housed in the perimeter of the oralobranchial chamber. This morphology is topologically consistent with placoderm braincases which show that the cucullaris muscle is serially aligned with the branchial levator muscles. Our investigation suggests it derived from the sixth branchial levator, consistent with the predictions of recent comparative developmental studies^29^. Despite the loss of posterior endoskeletal branchial arches in gnathostomes, a branchiomeric muscle of the sixth branchial arch (as the cucullaris muscle) maintained a consistent topological relationship with the dermal exoskeleton (Fig. 3). This new model of musculoskeletal transformation in pectoral girdle origins thus unifies a wide array of evidence on the origin of the pectoral girdle. It adds important new details to the biomechanical basis for the origin of the girdle and clarifies the comparative anatomy of key jawless and jawed fishes. This new framework is consistent with recent proposals of a dual origin of the pectoral girdle^16^ and thus contributes to the reconciliation of two long-debated theories of paired fin origins.

## Methods

### Additional specimen

We analysed an articulated specimen of *Romundina* from the Geowissenschaftliches Zentrum der Universität Göttingen, Museum & Collection (GZG) specimen 100–488A.

### Computed tomography scanning

We used x-ray computed microtomography to scan specimens of *Kolymaspis* and *Romundina*. We scanned the *Kolymaspis* specimen TsNIGR 7656 at the Natural History Museum (Imaging and Analysis Centre), UK using the Nikon Metrology HMX ST 225 system, 210 kV, 150 mA, 2.5 mm copper filter, and resulting voxel size of 70 µm. The *Romundina* specimen was scanned at the Cambridge University Museum of Zoology, using the Nikon Metrology HMX ST 225 system, 185 kV, 245 mA, 0.75 mm copper filter, and resulting voxel size of 43 µm.

### Osteostracan tomography

To explore osteostracan vascularization patterns, we created a tomographic image series from the digitized publication of Stensiö (1927)^33^. We used Series F of *Mimetaspis hoeli* in plates 106–112, cropping each slice using Adobe Photoshop and exporting each to a separate bitmap file. We then loaded the series into SPIERS Align^51^ and conducted a manual registration.

### Segmentation and surface model visualzation

We performed segmentation of the tomographic datasets using Materialise Mimics (https://www.materialise.com). We segmented *Kolymaspis* primarily using Mimics v. 18; we finalized and cleaned the masks using Mimics v. 24. We segmented *Mimetaspis* series F, *Romundina* specimen GZG 100–488A, and existing data of *Brindabellaspis* (Extended Data Fig. 1) using Mimics v. 25. We used cycles rendering in Blender (Blender Foundation, https://www.blender.org) to generate surface model images for publication-ready figures.

### Phylogenetic data

We used the dataset of King et al. ^43^ as the basis of our phylogenetic analysis, edited in Mesquite v 3.70^52^. It contains fully annotated character names and state labels. The removal of citation histories complicates dataset comparison and incorporation of new characters and data. Subsequent datasets have extinguished the records for many characters, resulting in incomplete character ontologies. This makes it nearly impossible for subsequent investigators to expand based on existing characters or to understand the original authors’ intentions. We generated a change log using a newly developed command line tool diffmatrix (https://github.com/mbrazeau/diffmatrix/releases/tag/v2.2).

To preserve character histories and ontologies, we did not permanently delete any taxa or characters from the matrix. Rather than deleting characters or taxa we considered problematic, we preserved the overall integrity of the matrix by using exclusion settings before phylogenetic analysis. Instead of attaching the phylogenetic data file as separate character list and matrix, we have stored it as a single Nexus file in a version-control archive (GitHub) as a way of improving maintainability (https://mbrazeau.github.io/gnathostome_characters). This also includes a link to a character list web page with character descriptions and citations. Changes to the matrix are detailed in the change log at https://mbrazeau.github.io/gnathostome_characters/changelog.html. A permanent version of the final dataset used in this study is archived at https://github.com/mbrazeau/gnathostome_characters/releases/tag/1.0 and a copy of the Nexus file is provided in the Supplementary Information.

### Phylogenetic analysis

We conducted a phylogenetic search using TNT (v. 1.6)^53^. We constrained the outgroup so that osteostracans and jawed vertebrates were each monophyletic. Because TNT constraints cannot conflict with outgroup choice, we later rerooted the trees so that Galeaspida were also monophyletic. Because all character types are symmetric this has no impact on the results. We set the tree buffer to 10,000 trees (hold 10000;) for unconstrained searches and up to 50,000 for searches under different constraints for placoderm monophyly. We conducted a ‘new technology search’ with the following command and settings to apply 50 iterations on the parsimony ratchet: xmult=level 10 ratchet 50; To further explore the resulting islands, we used additional branch-breaking (bbreak=fillonly;) to swap the trees in memory. We then computed the strict consensus tree using the nelsen command. We conducted two additional searches constraining placoderms to be monophyletic. The first constrained as monophyletic all placoderms inclusive of *Entelognathus* and *Minjinia*, while the second constrained only the ‘core’ placoderms^54^ (excluding *Entelognathus* and *Minjinia* from the placoderm constraint).

### Reporting summary

Further information on research design is available in the Nature Portfolio Reporting Summary linked to this article.

## Online content

Any methods, additional references, Nature Portfolio reporting summaries, source data, extended data, supplementary information, acknowledgements, peer review information; details of author contributions and competing interests; and statements of data and code availability are available at 10.1038/s41586-023-06702-4.

### Supplementary information

Supplementary Information This file contains additional specimen information (geological provenance and age), additional phylogenetic results, and supplementary references.

Reporting Summary

Supplementary Data An archive folder containing the original Nexus file (MATRIX_MASTER.nex), the exported TNT file, TNT script, and a README file for instructions on reproducing the phylogenetic analysis.

## Data Availability

Scan data and relevant surface meshes of *Kolymaspis* and *Romundina* are provided on FigShare (10.6084/m9.figshare.22579840). The character list is stored in the original Nexus file, including character descriptions and references. Readers can access the file at https://mbrazeau.github.io/gnathostome_characters/ or in the Supplementary Information. Changes to the matrix are detailed in the change log at https://mbrazeau.github.io/gnathostome_characters/changelog.html.

## Code availability

All TNT commands and scripts used in our analyses are provided in the Supplementary Information. Diffmatrix v2.2 used to generate the matrix change log is available as both source code and both macOS and Windows executables from https://github.com/mbrazeau/diffmatrix/releases/tag/v2.2.
