# Supplementary material for: Fossil evidence for a pharyngeal origin of the vertebrate pectoral girdle
Source: Nature. 2023 Nov 1;623(7987):550–4. doi: 10.1038/s41586-023-06702-4 (PMC10651482; doi:10.1038/s41586-023-06702-4)
Supplement: Supplementary file 1 — This file contains additional specimen information (geological provenance and age), additional phylogenetic results, and supplementary references. [file 41586_2023_6702_MOESM1_ESM.docx]

**Supplementary information for:**

**Fossil evidence for the pharyngeal origin of the vertebrate pectoral girdle**

Martin D Brazeau^1,2^, Marco Castiello^1,3^, Amin El Fassi El Fehri^1^, Louis Hamilton^1^, Alexander O Ivanov^4,5^, Zerina Johanson^2^, Matt Friedman^6^

^1^ Department of Life Sciences, Imperial College London; Silwood Park Campus, Buckhurst Road, Ascot, SL5 7PY, United Kingdom

^2^ The Natural History Museum; Cromwell Road, London, SW7 5BD, United Kingdom

^3^ London Academy of Excellence Lilywhite House, 780 High Rd, London N17 0BX, United Kingdom

^4^ Department of Sedimentary Geology, Institute of Earth Sciences, St. Petersburg State University, 16 Line 29, St. Petersburg 199178, Russia

^5^ Institute of Geology and Petroleum Technologies, Kazan Federal University, Kremlevskaya St., 4/5, Kazan 420008, Russia

^6^ Museum of Paleontology and Department of Earth and Environmental Sciences; North University Avenue, Ann Arbor, MI 48109, USA

*Corresponding author. Email: [m.brazeau@imperial.ac.uk](mailto:m.brazeau@imperial.ac.uk)

**Contents**

**1. Additional specimen information**

**2. Supplementary phylogenetic results**

**3. Supplementary references**

**1. Additional specimen information**

The holotype of *Kolymaspis sibirica* Bystrow, 1956 (TSNIGR 7656) was collected in the Lower Devonian Vechernyaya Formation of Taskan River, Kolyma River Basin, Omuliovka Uplift, North-eastern Siberia, Russia by Yu. N. Popov (Bystrow, 1956). The specimen was discovered in a layer of dark bituminous limestone which has been dated by conodonts and corresponds to the *kitabicus*– Lower *inversus* conodont zones of the Emsian stage (Gagiev, 1995)^55^.

**2. Supplementary phylogenetic results**

The equal-weights phylogenetic search filled the tree buffer of 10,000 topologies of length 1,884 steps. The implied weights search (k=12) resulted in 181 trees with a score of 42.64171. Strict consensus trees for both analyses are shown in Extended Data Fig. 5. Bootstrap analysis finds generally weak support for the overall arrangement of placoderms as paraphyletic, with lower than 50% bootstrap support for the node resolving *Kolymaspis* and *Brindabellaspis* to the exclusion of other jawed vertebrates.

Under the equal weights parsimony tree, character state optimisations indicate three unambiguous synapomorphies that support a clade consisting of all placoderms and crown-group jawed vertebrates to the exclusion of *Brindabellaspis* and *Kolymaspis*:

| No. | Character name | State change | Consistency index |
| --- | --- | --- | --- |
| 177 | Dermal cranio-thoracic articulation between paired main-lateral-line-bearing bones of skull and shoulder girdle | Absent 🡪 Present | 0.5 |
| 180 | Endoskeletal craniothoracic (sixth branchial) facet | Present 🡪 Absent | 1.0 |
| 251 | Preorbital depression | Absent 🡪 Present | 0.2 |

The constrained analyses both filled the tree buffers. The constraint on all placoderms resulted in a score of 1,191 steps. The “core” placoderm constraint analysis resulted in a score of 1,188 steps. In the all-placoderms constraint, *Kolymaspis* and *Brindabellaspis* are nested deeply inside the placoderms, as the sister group of antiarchs. In the core-constraint tree, three main placoderm clades (and two smaller subclades/branches) are resolved in a basal polytomy. The former result is inconsistent with the hypothesis that the endoskeletal shoulder link in these taxa is homologous with conditions in jawless outgroups. However, in the latter analysis the result is equivocal and depends on the resolution of the polytomy.

**3. Supplementary references**

55. Gagiev, M. H. Stratigraphiya devona i nizhnego karbona Omuliovskogo podnyatiya (Severo-Vostok Azii)[Stratigraphy of Devonian аnd Loweг Carboniferous of the Omuliovka Uplift (North-eastern Asia)]. Magadan: NEISRI FEB RAS, 196 р. (1995) [In Russian].
