# Supplementary material for: Fossil evidence for a pharyngeal origin of the vertebrate pectoral girdle
Source: Nature. 2023 Nov 1;623(7987):550–4. doi: 10.1038/s41586-023-06702-4 (PMC10651482; doi:10.1038/s41586-023-06702-4)
Supplement: Supplementary file 2 — Reporting Summary [file 41586_2023_6702_MOESM2_ESM.pdf]

nature portfolio

Corresponding author(s): Martin D Brazeau

Last updated by author(s): Sep 13, 2023

Reporting Summary

Nature Portfolio wishes to improve the reproducibility of the work that we publish. This form provides structure for consistency and transparency in reporting. For further information on Nature Portfolio policies, see our Editorial Policies and the Editorial Policy Checklist.

Statistics

For all statistical analyses, confirm that the following items are present in the figure legend, table legend, main text, or Methods section.

n/a	Confirmed	
☐	☒	The exact sample size (*n*) for each experimental group/condition, given as a discrete number and unit of measurement
☒	☐	A statement on whether measurements were taken from distinct samples or whether the same sample was measured repeatedly
☒	☐	The statistical test(s) used AND whether they are one- or two-sided<br>*Only common tests should be described solely by name; describe more complex techniques in the Methods section.*
☒	☐	A description of all covariates tested
☐	☒	A description of any assumptions or corrections, such as tests of normality and adjustment for multiple comparisons
☒	☐	A full description of the statistical parameters including central tendency (e.g. means) or other basic estimates (e.g. regression coefficient) AND variation (e.g. standard deviation) or associated estimates of uncertainty (e.g. confidence intervals)
☒	☐	For null hypothesis testing, the test statistic (e.g. *F*, *t*, *r*) with confidence intervals, effect sizes, degrees of freedom and *P* value noted<br>*Give P values as exact values whenever suitable.*
☒	☐	For Bayesian analysis, information on the choice of priors and Markov chain Monte Carlo settings
☒	☐	For hierarchical and complex designs, identification of the appropriate level for tests and full reporting of outcomes
☒	☐	Estimates of effect sizes (e.g. Cohen's *d*, Pearson's *r*), indicating how they were calculated

Our web collection on statistics for biologists contains articles on many of the points above.

Software and code

Policy information about availability of computer code

Data collection	We performed segmentation of the tomographic datasets using Materialise Mimics (https://www.materialise.com). We segmented Kolymaspis was primarily using Mimics v. 18; we finalized and cleaned the masks using Mimics v. 24. We segmented Mimetaspis Series F and Romundina specimen GZG 100-488A using Mimics v. 25. We used cycles rendering in Blender 3.2.2 (Blender Foundation, https://www.blender.org) to generate surface model images for publication-ready figures. We used SPIERS Align v. 3.1 (Sutton et al.) and conducted a manual registration.
Data analysis	We conducted a phylogenetic search using TNT (v. 1.5) (Goloboff & Catalano 2015). We wrote a custom software tool diffmatrix v2.2 to compare our dataset to an earlier iteration. This has been made available through a link to GitHub: https://github.com/mbrazeau/diffmatrix (release version: https://github.com/mbrazeau/diffmatrix/releases/tag/v2.2)

For manuscripts utilizing custom algorithms or software that are central to the research but not yet described in published literature, software must be made available to editors and reviewers. We strongly encourage code deposition in a community repository (e.g. GitHub). See the Nature Portfolio guidelines for submitting code & software for further information.

Data

Policy information about availability of data

All manuscripts must include a data availability statement. This statement should provide the following information, where applicable:

- Accession codes, unique identifiers, or web links for publicly available datasets
- A description of any restrictions on data availability
- For clinical datasets or third party data, please ensure that the statement adheres to our policy

Scan data and relevant surface meshes of Kolymaspis and Romundina are provided deposited on FigShare (10.6084/m9.figshare.22579840). The character list is stored in the original Nexus file including character descriptions and references. Readers can access the file at https://mbrazeau.github.io/gnathostome_characters/ which also includes a link to a character list web page. Changes to the matrix are detailed in the change log at https://mbrazeau.github.io/gnathostome_characters/changelog.html . A permanent version of the final dataset is archived at (https://github.com/mbrazeau/gnathostome_characters/releases/tag/1.0-review).

Research involving human participants, their data, or biological material

Policy information about studies with human participants or human data. See also policy information about sex, gender (identity/presentation), and sexual orientation and race, ethnicity and racism.

Reporting on sex and gender	n/a
Reporting on race, ethnicity, or other socially relevant groupings	n/a
Population characteristics	n/a
Recruitment	n/a
Ethics oversight	n/a

Note that full information on the approval of the study protocol must also be provided in the manuscript.

Field-specific reporting

Please select the one below that is the best fit for your research. If you are not sure, read the appropriate sections before making your selection.

☐ Life sciences ☐ Behavioural & social sciences ☒ Ecological, evolutionary & environmental sciences

For a reference copy of the document with all sections, see nature.com/documents/nr-reporting-summary-flat.pdf

Ecological, evolutionary & environmental sciences study design

All studies must disclose on these points even when the disclosure is negative.

Study description	Anatomical descriptions of existing fossil material using computed tomography. This was supplemented with literature-based and collections-based comparative analysis.
Research sample	The main focus of this work was the braincase of the enigmatic placoderm fish, Kolymaspis sibirica from the Early Devonian of Siberia. This placoderm fish reveals a unique morphology of the head-shoulder linkage which provides clues to the relationship between the pharynx and shoulder in early gnathostomes.
Sampling strategy	n/a
Data collection	n/a
Timing and spatial scale	n/a
Data exclusions	n/a
Reproducibility	All specimens involved in this study are housed in permanent repositories. We have furthermore provided a complete digital archive of all relevant data required to reproduce the study.
Randomization	n/a
Blinding	n/a

Did the study involve field work? ☐ Yes ☒ No

Reporting for specific materials, systems and methods

We require information from authors about some types of materials, experimental systems and methods used in many studies. Here, indicate whether each material, system or method listed is relevant to your study. If you are not sure if a list item applies to your research, read the appropriate section before selecting a response.

Materials & experimental systems

n/a	Involved in the study
☒	☐ Antibodies
☒	☐ Eukaryotic cell lines
☐	☒ Palaeontology and archaeology
☒	☐ Animals and other organisms
☒	☐ Clinical data
☒	☐ Dual use research of concern
☒	☐ Plants

Methods

n/a	Involved in the study
☒	☐ ChIP-seq
☒	☐ Flow cytometry
☒	☐ MRI-based neuroimaging

Palaeontology and Archaeology

Specimen provenance: Kolymaspis sibirica is from the Magadan Oblast of far eastern Siberia. It was collected in the mid-20th century. The specimen of Romundina is from the Drake Bay formation on Prince of Wales Island, in what is now Nunavut. It was collected in 1975 by Hans-Peter Schultze and Frank Langenstrassen when the region was still part of the Northwest Territories.

Specimen deposition: Kolymaspis is deposited in the FN Chernyshev Central Research Geological Museum in St. Petersburg, Russia. Romundina specimen is housed in Geowissenschaftliches Zentrum der Universität Göttingen, Museum & Collection (GZG)

Dating methods: n/a

☐ Tick this box to confirm that the raw and calibrated dates are available in the paper or in Supplementary Information.

Ethics oversight: No ethical approvals were required as all material is housed in existing accredited collections .

Note that full information on the approval of the study protocol must also be provided in the manuscript.
